# Identification of three predictors of gastric cancer progression and prognosis

**DOI:** 10.1002/2211-5463.12943

**Published:** 2020-08-18

**Authors:** Kai Huang, Shuhua Chen, Rongzhang Xie, Pengpeng Jiang, Changjun Yu, Jinmei Fang, Xingcun Liu, Fazhi Yu

**Affiliations:** ^1^ Department of Gastrointestinal Surgery Department of General Surgery the First Affiliated Hospital of Anhui Medical University Hefei China; ^2^ Department of Laboratory Medicine Yunfu People's Hospital Southern Medical University Yunfu China; ^3^ Department of Radiotherapy Anhui Provincial Hospital the First Affiliated Hospital of the University of Science and Technology of China Hefei China; ^4^ School of Life Sciences University of Science and Technology of China Hefei China

**Keywords:** gastric cancer, potential predictors, TCGA, GEO

## Abstract

Abnormal gene expression is an established cause of gastric cancer (GC) initiation and progression. In this study, we aimed to identify several key genes that could be used to effectively predict progression and prognosis in patients with GC. The Cancer Genome Atlas and the Gene Expression Omnibus database were used to identify candidate genes. Fourteen genes were found to associate highly with progress, metastasis, and survival of GC. Five of these genes were overexpressed in tumor tissue compared to adjacent normal tissue. This was confirmed by reverse transcription‐polymerase chain reaction and western blotting for myosin‐Va (MYO5A), phospholipid transfer protein (PLTP), and tripeptidyl peptidase 1 (TPP1), while the CCK8 assay was used to show that these three genes promote GC cell proliferation. In summary, we demonstrate that MYO5A, PLTP, and TPP1 expression may be suitable markers for the progression and prognosis of GC.

## Graphical abstract

Abnormal gene expression is an established cause of gastric cancer (GC) initiation and progression. By integrating bioinformatics and assay validations, we demonstrate that high expression of MYO5A, PLTP, or TPP1 is associated with tumor progress (1) and poor prognosis (2) in GC patients. These three genes may have potential to serve as predictive biomarkers for GC diagnosis and treatment.

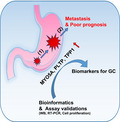

**Abbreviations**
CCK8: cell counting Kit‐8
GC: gastric cancer
GEO: Gene Expression Omnibus
MYO5A: myosin‐Va
PLTP: phospholipid transfer protein
RT–PCR: reverse transcription‐polymerase chain reaction
TCGA: The Cancer Genome Atlas
TPP1: tripeptidyl peptidase 1

Gastric cancer (GC) remains one of the most commonly occurring gastrointestinal tumors worldwide. According to Global Cancer Statistics 2018, it is the second leading cause of cancer‐related mortality and has a substantial global economic burden [1]. In China, 679 100 people were newly diagnosed with GC and 498 000 died in 2015 alone [2]. The symptoms of GC are not obvious during the early stages, but when they become apparent, GC patients have usually reached an advanced stage and the tumor has already metastasized [3]. Surgical intervention and chemotherapy are the main therapeutic strategies for advanced GC; however, despite improvements in these areas, the prognosis of GC patients remains poor [4, 5]. This is mainly due to lack of sensitive and specific predictors for GC diagnosis. Therefore, biomarkers for early and accurate diagnosis of GC are urgently needed.

With rapid advances in genome sequencing technology, RNAseq results are being increasingly used to aid clinical diagnosis and treatment of cancer [6]. Biological and molecular alterations underlying onset and progression of GC can be comprehensively analyzed from genome sequencing data. Integrating this information with clinical data could help predict the progress and prognosis of GC.

In this study, we aimed to identify genes that could serve as biomarkers in gastric patients. Based on The Cancer Genome Atlas (TCGA) data in LinkedOmics [7], as well as GSE118916 [8] and GSE54129 datasets from the Gene Expression Omnibus (GEO) database, we identified myosin‐Va (MYO5A), tripeptidyl peptidase 1 (TPP1), TGFBR2, PALM2‐AKAP2, and PLTP as potential predictors of progression and prognosis of GC. Clinical samples from GC patients were used to confirm the mRNA and protein expression of these five biomarker genes. We report that MYO5A, PLTP, and TPP1 were more expressed in tumors than in adjacent tissue specimens and were involved in promoting GC cell proliferation *in vitro*. In conclusion, our work can help clinicians formulate personalized treatment and exempt patients from unnecessary exposure to chemotherapy.

## Materials and methods

### Data extraction from TCGA‐Stomach cancer

The association between mRNA expression and GC pathological stage, number of metastatic lymph nodes, and survival in patients with primary GC in TCGA‐Stomach cancer was analyzed using the LinkedOmics browser (http://www.linkedomics.org) [7], which allows the online analysis of TCGA data.

### Data extraction from the GEO database

The gene expression profiles of two GEO datasets, GSE118916 (*n* = 30) [8] and GSE54129 (*n* = 132) (B. Liu, Z. Zhu, M. Yan, J. Li, J. Zhang & C. Li, unpublished data, 2014), with normal and gastric tumor samples were downloaded from the GEO DataSets database (https://www.ncbi.nlm.nih.gov/geo/).

GEO2R (https://www.ncbi.nlm.nih.gov/geo/geo2r/), an interactive online tool for analyzing two or more sample groups in a GEO Series, was used to detect differentially expressed genes between normal and GC samples according to the tool's manual [9].

### The Kaplan–Meier plotter

The association between expression of the five biomarker genes and overall survival (OS) was assessed by the Kaplan–Meier plotter (http://kmplot.com/analysis/), an online database that enables cross‐validation of survival‐associated biomarkers in GC [10]. GEO data were used, and patients were split according to the median expression of the target gene. For cutoff value definition, the autoselect best cutoff pattern was chosen and only the Jetset best probe of the gene was selected. For array quality control, we excluded biased arrays. The hazard ratio (HR) and log‐rank *P* value were calculated.

### Patients and samples

Twenty pairs of fresh GC and adjacent normal tissues from GC patients, who were diagnosed with GC in the First Affiliated Hospital of Anhui Medical University, were included in the study. The study was approved by the Ethics Committee of Anhui Medical University (Anhui, China) and conformed to the guidelines set by the Declaration of Helsinki. All patients who participated in this study provided written informed consent. The identity of all GC samples and normal gastric tissues was confirmed by histopathological analysis, which revealed also that adenocarcinoma was the pathological type of GC. Tissue fragments were frozen in liquid nitrogen immediately after surgical excision. Total protein and RNA from tissue samples were extracted and stored at −80 °C. Detailed patient information is provided in Table 1.

**Table 1:** Patients' information.

Patients’ No.	Gender	Age (years)	Histological grade	Lauren's classification	Clinical stage	Lymph node metastasis
1	Male	35	G1	Diffuse	Ⅰ	No
2	Female	69	G3	Intestinal	Ⅲ	Yes
3	Male	48	G1	Diffuse	Ⅰ	No
4	Male	55	G2	Diffuse	Ⅱ	No
5	Female	58	G1	Intestinal	Ⅰ	No
6	Female	42	G1	Diffuse	Ⅰ	Yes
7	Male	66	G2	Intestinal	Ⅱ	No
8	Male	53	G3	Diffuse	Ⅲ	Yes
9	Female	47	G3	Diffuse	Ⅳ	Yes
10	Male	43	G1	Diffuse	Ⅰ	Yes
11	Female	50	G2	Diffuse	Ⅱ	No
12	Female	70	G1	Intestinal	Ⅰ	No
13	Male	62	G2	Diffuse	Ⅱ	No
14	Male	44	G1	Diffuse	Ⅰ	Yes
15	Female	72	G3	Intestinal	Ⅲ	No
16	Female	68	G3	Intestinal	Ⅲ	Yes
17	Male	60	G1	Intestinal	Ⅰ	Yes
18	Male	55	G2	Diffuse	Ⅱ	Yes
19	Male	56	G1	Diffuse	Ⅰ	Yes
20	Female	66	G2	Intestinal	Ⅱ	Yes

### Cell culture and transfection

Gastric carcinoma cell lines AGS and SGC‐7901 were obtained from the American Type Culture Collection (Manassas, VA, USA) and were authenticated by short tandem repeat genotyping prior to use in experiments. The cell lines were maintained in Dulbecco's Modified Eagle's medium (Gibco, Grand Island, NY, USA) supplemented with 10% FBS (Gibco) in a humidified incubator with 5% CO_2_ at 37 °C. Mycoplasma contamination was examined routinely using a PCR mycoplasma detection kit. MYO5A‐specific siRNA (5′‐GAAUGUUCUGGAGAAAUUAGU‐3′), PLTP‐specific siRNA (5′‐GGACCUUCGAAGGUUUCAATT‐3′), and a negative control siRNA were purchased from Guangzhou RiboBio (Guangzhou, China). The siRNA transfection was achieved using Lipofectamine 3000 reagent (Invitrogen, Carlsbad, CA, USA) according to the manufacturer's instructions. After 72 h of incubation, the following *in vitro* experiments (see Sections RNA extraction and RT‐PCR, Western blot and CC K8 assay) were conducted.

### RNA extraction and RT–PCR

Total RNA was extracted from tissues and GC cell lines using TRIzol reagent (Thermo Fisher Scientific, Waltham, MA, USA) according to the manufacturer's instructions. Reverse transcription was performed using the cDNA Reverse Transcription Kit (Thermo Fisher Scientific). reverse transcription‐polymerase chain reaction (RT–PCR) amplification was performed using SYBR Premix Ex Taq (Takara, Beijing, China) in the Applied Biosystems 7500 Fast Real‐Time PCR System (Thermo Fisher Scientific). The sequences of the sense and antisense primers are listed in Table 2. The relative amount of gene normalized to the control was calculated with equation $2^{‐\Delta C_{t}}$. All the reactions were run in triplicate.

**Table 2:** Primers and antibodies used in this work.

Gene names	Forward	Reverse	Antibody
MYO5A	ATCTCCTGCTACCTATGT	CCACGAATACTCTGACTT	ab235003
PPT1	GACCTGTAGCTTGATCACCTCA	GAACGCACATCTATGGGAGC	ab96498
TGFBR2	ACTGCCCATCCACTGAGACAT	CCATACAGCCACACAGACTTCC	ab184948
PALM2‐AKAP2	GATGAAGGACAGTGGTGATA	CAGTGAAGAGAATAAGCAGAC	ab64904
PLTP	CGTGCGTAGTTCTGTGGATG	CATCCTCTCGTCGTCATCCA	ab134066
GAPDH	TGCACAGGAGCCAAGAGTGAA	CACATCACAGCTCCCCACCA	ab8245

### Western blot

Total protein was extracted using RIPA buffer (Beyotime, Shanghai, China), and the concentration was measured using the bicinchoninic acid assay (Sangon Biotech, Shanghai, China). Proteins were separated by 10% SDS/PAGE and then transferred to polyvinylidene difluoride membranes. After blocking with 5% nonfat milk, the membrane was incubated with primary antibodies (Abcam, Cambridge, UK; see Table 2) at 4 °C overnight. Afterward, the membrane was washed with TBST and incubated with secondary antibody conjugated with horseradish peroxidase for 2 h at room temperature. Protein bands were visualized using enhanced chemiluminescence (Pierce, Rockford, IL, USA). Glyceraldehyde 3‐phosphate dehydrogenase (GAPDH) was used as a loading control. And the intensity of each band was qualified using imagej (NIH, Bethesda, MD, USA).

### CCK8 assay

The cell proliferation ratio was detected using the CCK8 kit (Beyotime). At 72 h after transfection, cells were transferred to 96‐well culture plates according to the kit's manual. Optical density at 490 nm was detected from day 1 (the day after cell transfer) to day 4.

### Statistical analysis

graphpad prism (GraphPad Software Inc, San Diego, CA, USA) and r language（A free software, Version 3.6.3） were used to analyze the data. *P* < 0.05 was considered statistically significant.

## Results

### Strategy for the identification of potential biomarker genes for GC

**Fig. 1:**
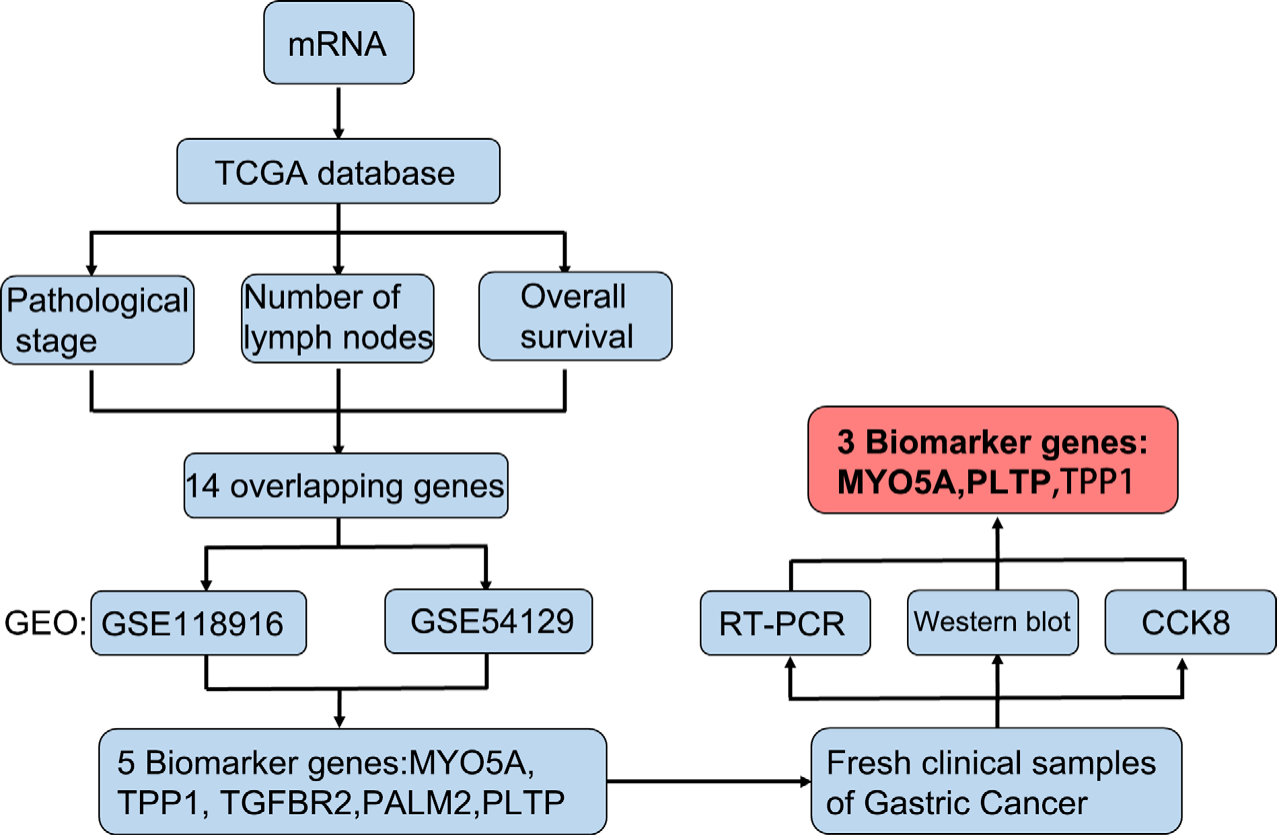
Experimental workflow for the identification of potential biomarker genes of GC. Data from TCGA and the GEO database were used to identify the genes that could be used to accurately predict the progression and prognosis of patients with GC.

As shown in Fig. 1, analysis of TCGA data revealed 14 genes, whose expression was significantly positively associated with pathological stage, number of metastatic lymph nodes, and OS. The expression of these genes in GC and adjacent normal tissues was analyzed using GEO datasets. Five genes, MYO5A, TPP1, TGFBR2, PALM2‐AKAP2, and PLTP, were expressed more in tumors than in normal tissues. RT–PCR, western blotting, and the CCK8 assay confirmed that MYO5A, PLTP, and TPP1 exhibited higher expression in GC tissue and were involved in promoting GC cancer cell proliferation.

### Identification of 14 genes associated with the progression of stomach adenocarcinoma

**Fig. 2:**
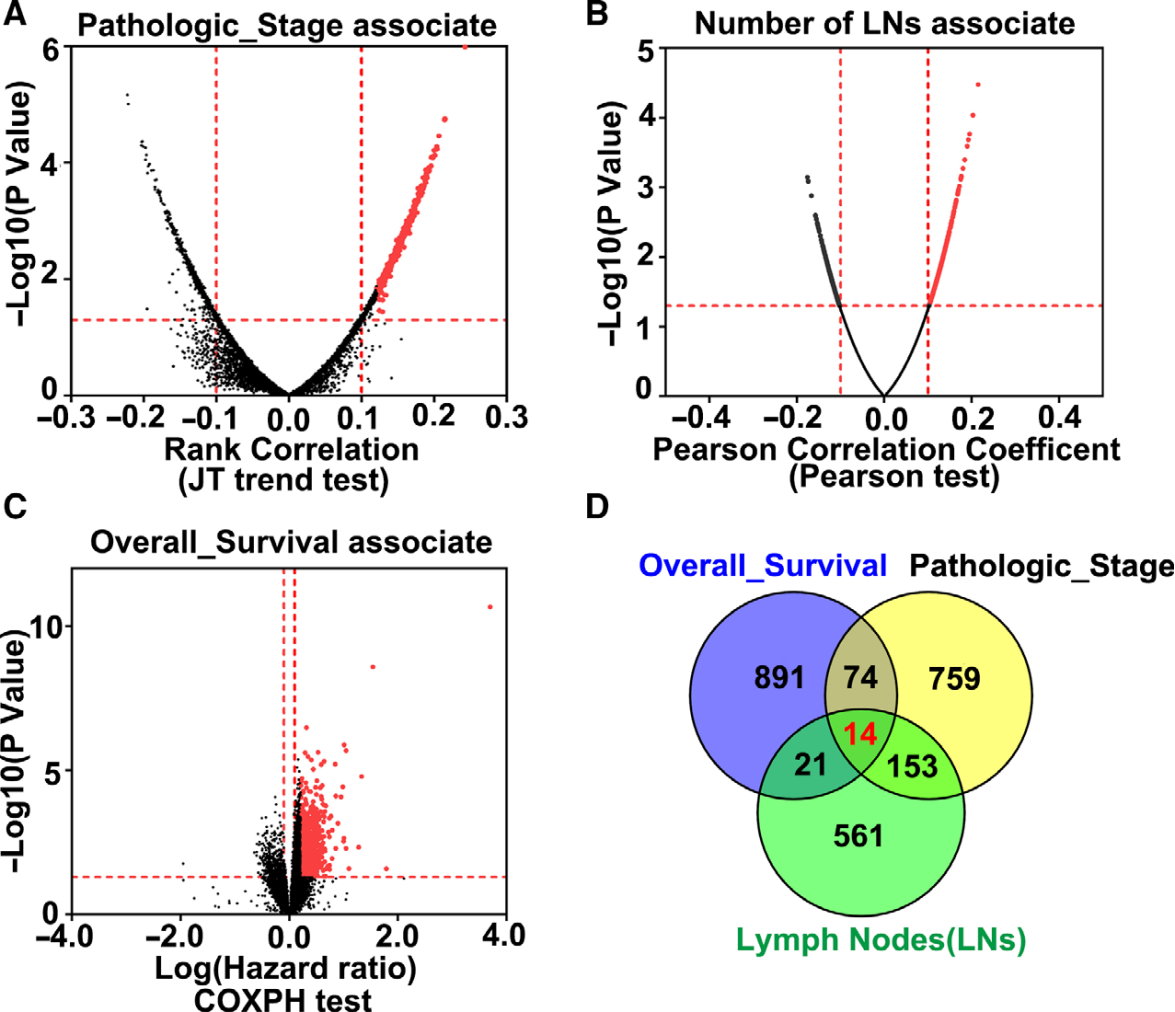
Fourteen genes are associated with the progression of stomach adenocarcinoma. (A‐C) Volcano map of the genes related to the pathological stage (A), the number of metastatic lymph nodes (B), and OS (C) of GC. (D) Venn diagram showing overlap between three different groups of genes positively associated with the pathological stage, number of metastatic lymph nodes, and OS of GC.

Tumor progression is driven mainly by abnormal gene expression. We hypothesized that if gene expression was associated with the pathological stage, the number of metastatic lymph nodes, and OS of cancer, then this gene could be used as a reliable biomarker to predict the progression and prognosis of GC. To identify the biomarker genes, we analyzed the TCGA data of GC. Figure 2 shows differentially expressed genes associated with the pathological stage (Fig. 2A), the number of metastatic lymph nodes (Fig. 2B), and OS (Fig. 2C). To obtain the overlapping genes, we selected the top 1000 genes that were significantly positively associated with pathological stage (Rank correlation > 0.12, *P* < 0.05) and OS (log(HR) > 0.21, *P* < 0.05), as well as the top 750 genes (Pearson correlation coefficient > 0.1, *P* < 0.05) significantly positively associated with the number of metastatic lymph nodes. Fourteen genes were found to associate positively with these three different factors (Fig. 2D).

### Expression of the candidate 14 genes in gastric tumor and adjacent normal tissue

**Fig. 3:**
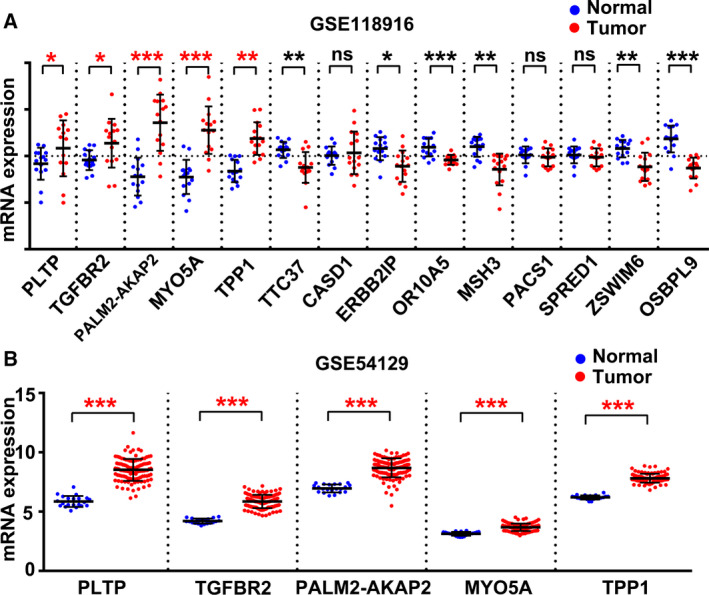
Analysis of the candidate 14 genes' expression in gastric tumors and adjacent normal tissue based on GSE118916 and GSE54129 datasets. (A) mRNA expression of genes in the GSE118916 dataset. Values are presented as the mean ± SD and were analyzed using a paired *t*‐test; *n* = 15/group. (B) mRNA expression of genes in the GSE54129 dataset. Data are presented as the mean ± SD and were analyzed using an unpaired *t*‐test; for normal tissues, *n* = 21, for tumor tissues, *n* = 111. *P < 0.05, **P < 0.01, ***P < 0.001.

The ideal biomarker gene should show high expression and be easily detected by common techniques such as immunohistochemistry. To this end, we compared expression of the 14 candidate genes in tumor tissue and adjacent normal tissues based on microarray data stored in the GSE118916 GEO dataset. As shown in Fig. 3A, mRNA expression of five genes, MYO5A, TPP1, TGFBR2, PALM2‐AKAP2, and PLTP, was significantly higher in GC tissue than in normal tissue (*P* < 0.05). Three genes showed no difference in expression between normal and tumor tissue, whereas for six genes, expression was significantly downregulated in tumor tissue. These results led us to hypothesize that MYO5A, TPP1, TGFBR2, PALM2‐AKAP2, and PLTP could be used as biomarker genes. To verify the results, we analyzed mRNA expression of the above five genes in the GSE54129 GEO dataset. As shown in Fig. 3B, these genes were confirmed to have higher expression in tumor tissue than in normal tissue. Taken together, MYO5A, TPP1, TGFBR2, PALM2‐AKAP2, and PLTP appear to be candidate predictors of GC.

### Validation of the expression of potential biomarker genes using GC patients’ samples

**Fig. 4:**
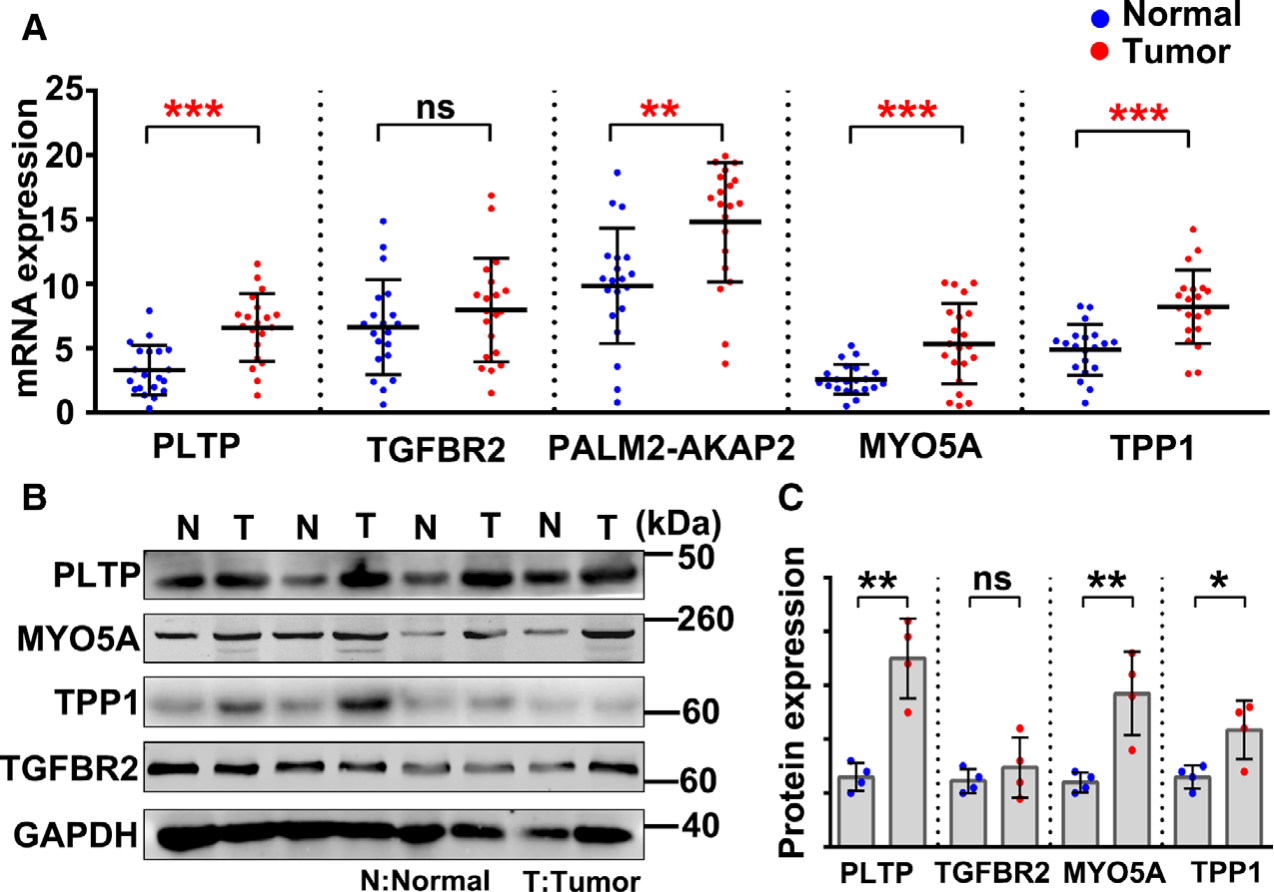
mRNA and protein expression of the biomarker genes. (A) RT–PCR results showing mRNA expression of the biomarker genes in human gastric tumor tissue and adjacent normal tissue specimens. Data are presented as the mean ± SD and were analyzed using a paired *t*‐test; *n* = 20/group. (B) Western blot results showing protein expression of the biomarker genes in tumor tissue and adjacent normal tissue specimens; *n* = 20/group. (C) Relative protein expression of the biomarker genes. The intensity of each band in B was quantified using imagej, and the loading control was used as reference. Data are presented as the mean ± SD and were analyzed using a paired *t*‐test; *n* = 4/group.*P < 0.05, **P < 0.01, ***P < 0.001.

To determine whether the above‐identified five genes could be used as diagnosis and prognosis biomarkers for GC, we collected 20 pairs of fresh tumor and adjacent normal tissue specimens from GC patients. RT–PCR confirmed higher expression of MYO5A, PLTP, PALM2‐AKAP2, and TPP1 in tumor tissue than in normal tissue specimens (Fig. 4A). Western blotting confirmed that protein expression of MYO5A, PLTP, and TPP1 was higher in tumor tissue than in normal tissue specimens (Fig. 4B,C). These results confirm that MYO5A, PLTP, and TPP1 are highly detectable predictive biomarkers of GC.

### MYO5A and PLTP promote GC cell proliferation

**Fig. 5:**
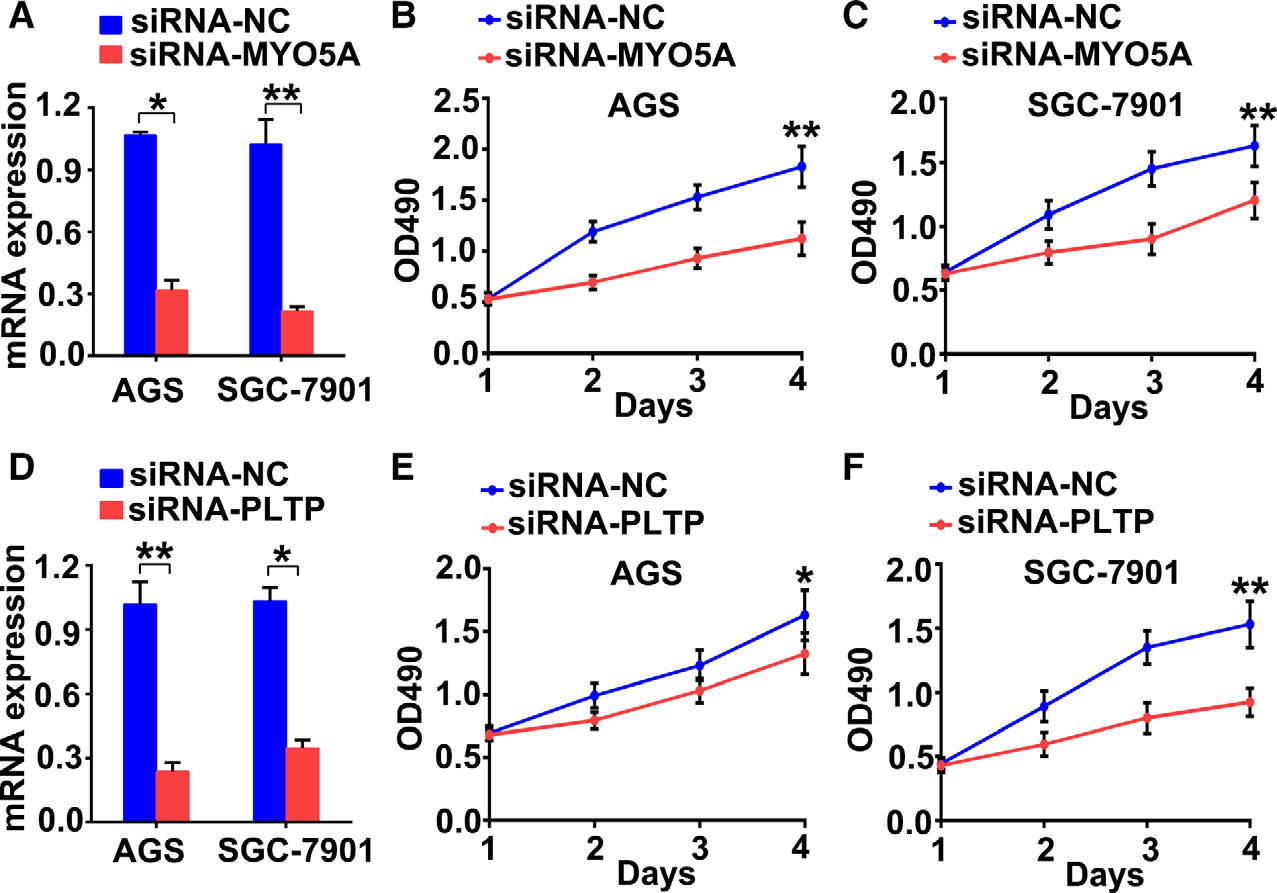
MYO5A and PLTP promote GC cell proliferation. (A) RT–PCR results showing knockdown efficiency of MYO5A. (B‐C) Cell proliferation rates detected using the CCK8 assay in AGS (B) and SGC‐7901 (C) cell lines. (D) RT–PCR results showing the knockdown efficiency of PLTP. (E‐F) Cell proliferation rates detected using the MTT assay in AGS (E) and SGC‐7901 (F) cell lines. *P < 0.05, **P < 0.01. Data are presented as the mean ± SD and were analyzed using an unpaired *t*‐test; *n* = 4/group.

Next, we examined the function of the biomarker genes. The CCK8 assay was performed to detect cell proliferation after knockdown of MYO5A or PLTP (Fig. 5). As shown in Fig. 5A,D, both MYO5A and PLTP were effectively knocked down and cell proliferation was inhibited in both AGS and SGC‐7901 cell lines (Fig. 5B,C and E,F, respectively). These findings demonstrate that MYO5A and PLTP are involved in promoting GC cell proliferation.

### Survival curve of the three potential biomarker genes

**Fig. 6:**
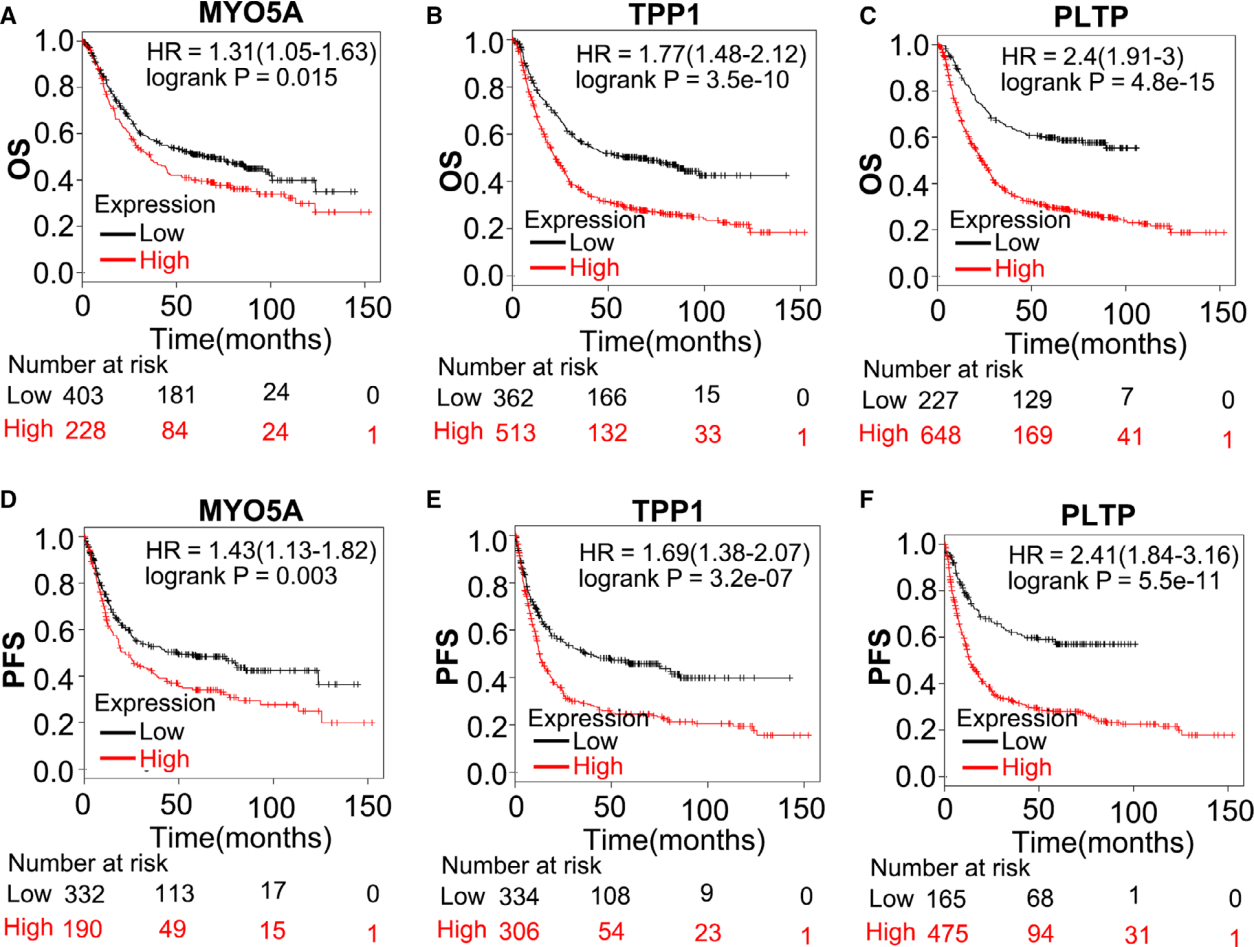
Survival analysis using RNAseq datasets in the Kaplan–Meier plotter. (A‐F) OS and PFS curves of MYO5A (A, D), TPP1 (B, E), and PLTP (C, F).

To confirm that expression of the three genes was positively associated with survival of GC patients, we analyzed the GC GEO data with the Kaplan–Meier plotter. As shown in Fig. 6, MYO5A, TPP1, and PLTP were significantly associated with OS or progression‐free survival (PFS) of GC. Altogether, we suggest that MYO5A, TPP1, and PLTP could be used as biomarkers for the diagnosis and prognosis of GC.

## Discussion

Although the morbidity of GC has seen a decline in recent years in China, it remains the second leading cause of death among cancer patients [2]. This loss of lives is mainly due to the impossibility of early screening and diagnosis of GC. Therefore, sensitive and specific molecular biomarkers of GC are urgently needed. Progress in RNA sequencing technology has been accompanied by the identification of new cancer prognostic signatures [11, 12, 13, 14]. Using genes as molecular biomarkers has attracted increasing attention because this method is useful for tracking the pathogenesis of GC.

In this work, we used TCGA data to screen genes that were positively associated with GC progress. Five of the 14 identified genes, including MYO5A, TPP1, TGFBR2, PALM2‐AKAP2, and PLTP, displayed higher expression in GC tumor tissue than adjacent normal tissue. We propose that the expression of these five genes can serve as a predictor of GC progress and prognosis. Using gastric tumor and adjacent normal tissue specimens collected in our hospital, we showed that MYO5A, PLTP, and TPP1 exhibited higher mRNA and protein expression in tumors than in normal tissue.

MYO5A is an actin‐dependent motor protein essential for the intracellular transport of organelles [15]. MYO5A plays an important role in malignant melanoma [16], and its expression was found to be elevated in a number of highly metastatic cancer cell lines and metastatic colorectal cancer tissues [17]. Moreover, overexpression of MYO5A is associated with neck lymph nodes metastasis of oral squamous cell carcinoma [18]. Importantly, overexpression of serum MYO5A in laryngeal squamous cell carcinoma predicted cervical nodal occult metastasis and poor prognosis [19].

The telomere‐binding POT1‐interacting protein (TPP1) is involved in protecting telomeres [20]. Previous studies reported that suppression of TPP1 caused telomere dysfunction and enhanced radiation sensitivity in a telomerase‐negative osteosarcoma cell line [21]. TPP1 was shown to modulate also telomere homeostasis and confer radioresistance to human colorectal cancer cells [22].

Phospholipid transfer protein (PLTP) is a widely expressed lipid transfer protein. It plays important roles in plasma lipoprotein metabolism [23] and transfers phospholipids from triglyceride‐rich lipoproteins to high‐density lipoprotein [24]. Overexpression of PLTP protein could promote growth and migration of human glioma cells [25].

In this study, we report that MYO5A, PLTP, and TPP1 exhibited higher expression in GC than in normal tissues, and this elevated expression associated positively with GC progress and prognosis. Moreover, we demonstrate that these genes act as oncogenes in GC cell lines because they promoted GC cell proliferation. Future work should examine whether these genes are involved in regulating GC metastasis and cell proliferation *in vitro* and *in vivo*, as well as how they exert their vital functions in GC.

## Conclusion

In conclusion, this study demonstrates that a high expression of MYO5A, PLTP, or TPP1 is associated with tumor progress and poor prognosis in GC patients. These three genes have significant potential to serve as predictive biomarkers for GC diagnosis and treatment.

## Conflict of interest

The authors declare no conflict of interest.

## Author contributions

KH and FY contributed to the design and conception of the study. SC and RX collected patients' samples and performed RT–PCR, western blot, and the CCK8 assay. KH, PJ, JF, and FY carried out data acquisition, analysis, and interpretation. KH and FY drafted the manuscript. CY and XL oversaw critical revision of the manuscript for intellectual content.

## Data Availability

The original data will be available from the corresponding author upon reasonable request.
